# Who is afraid of emergency politics? Public opinion on European crisis management during Covid-19

**DOI:** 10.1057/s41295-023-00329-5

**Published:** 2023-02-06

**Authors:** Joseph Ganderson, Waltraud Schelkle, Zbigniew Truchlewski

**Affiliations:** European Institute, London School of Economics and Political Science, Houghton Street, London, WC2A 2AE UK

**Keywords:** Crisis politics, Emergency politics, Covid-19, European Union politics, Public opinion, Cleavage theory

## Abstract

After a decade of crisis management, the democratic implications of emergency modes of governance in the European Union (EU) are under the spotlight. The prevailing analysis is critical. Scholars point to an emergent, distinctly European trend of transnational crisis exploitation where elite appeals to exceptional pressures serve asymmetric power and influence, overriding democratic norms and potentially fuelling Eurosceptic backlash. However, the literature does not ask whether citizens consider themselves disempowered by the EU’s emergency politics, with its alleged emphasis on urgency and technocratic problem-solving. The relative symmetry and simultaneity of the Covid-19 crisis across Europe offers an opportunity for an empirical examination of public opinion on traits of emergency politics. We juxtapose the implications of emergency politics for public opinion with the transnational cleavages literature and use survey data from 15 member states on EU- and national-level pandemic responses to examine the competing hypotheses. Our findings indicate perceptions of crisis management are largely determined by prior views on EU integration and democracy. More generally, the results suggest that the transnational cleavage remains overall a key driver and delimiter of Euroscepticism in crisis times. Though there is some variance between emergency politics dimensions, we do not detect a widespread perception of disillusionment motivated by EU emergency rule.

**Supplementary Information:**

The online version contains supplementary material available at 10.1057/s41295-023-00329-5.

## Voter perceptions of crisis management

It has become commonplace to describe the 2010s as the decade of the European Union’s (EU) ‘poly-crisis’ (Zeitlin et al. 2019, 973). Its responses to acute challenges, including the Euro-area and migration crises, have taken on a broadly integrative thrust, apparently validating Jean Monnet’s dictum that “Europe will be forged in crisis” (Monnet 1976, 488). The EU’s governance in crisis mode over an extended period has also attracted scrutiny by critics (Rhinard 2019; White 2015). They claim that the recourse of executive policymaking to exceptional circumstances undermines democratic procedures. The emergence of a distinctive mode of EU ‘emergency politics’ is said to be novel, uniquely transnational in its scope, and exerting a potentially deleterious effect on the future of the Union.^1^

Leading chroniclers of ‘emergency politics’ in the EU context define the term as “actions breaking with established norms and rules that are rationalised as necessary responses to exceptional and urgent threats” (Kreuder-Sonnen and White 2022, 954). Of course, criticism of the abuse of crisis events is well-established in ancient and contemporary political thought, couched typically at the level of executive dominance in nation states (Honig 2009). New is the claim of an apparently permanent “*transnational* state of exception” that merits special attention (Kreuder-Sonnen 2016, 1350; emphasis added). The critique of emergency politics raises similar concerns to the ‘democratic deficit’, a more longstanding complaint around EU governance vis-à-vis national democracy (Follesdal and Hix 2006). While national emergency politics is guided by a “defined sovereign”, in the EU it is said to be “informally co-produced by the many”, shaped by “structural factors beyond the sovereignty of individual states” (White 2019, 75, 78). This, in turn, is said to reinforce asymmetric power relations and override the accountability of democratic decision-making.

The gravity of this claim merits careful consideration (Oana et al. 2023). Independent of whether one considers EU crisis policymaking to be undermining representative deliberation (Rauh 2022; Truchlewski et al. 2021), the *effect* of this mode of policymaking on mass public perceptions of democratic politics in the EU is surely meaningful. If citizens find it problematic, EU crisis policymaking might breed distrust, fuelling Eurosceptic backlash. As Kreuder-Sonnen and White (2022, 961) state, “emergency politics fosters an anti-system politics in its own image”. This is a causal claim, linking ‘doing’ emergency politics with currents in public opinion. As such, it is surprising that scholarship concerned with democratic theory has not unpacked how the public perceives EU emergency politics in action. Showing little interest in gauging mass democratic sensitivities might amount to an ironic methodological elitism.^2^ An exception is White (2019), who postulates an explicit link from emergency politics to populist gains, but his is not a study in public opinion. He depicts challenger movements on the nationalist right as canaries in the coalmine, possibly indicative of a more widespread alienation with democratic representation in EU decision-making.

This contrasts with the literature on the new ‘transnational cleavage’, which is particularly concerned with public opinion and largely tells a reverse story: underlying socio-economic transformations shape attitudes towards transnational Europe (Kriesi et al. 2008; Hooghe and Marks 2009, 2018). Attitudes towards transnationalism are rooted in material interests and ideological pre-dispositions; hence, they are determinants of public opinion on EU governance rather than responses to it. By suggesting that governance might itself foster alienation, White’s argument is a plausible but radical departure, and one that clearly merits further empirical enquiry.

This article brings these two contemporary theories of EU politics into conversation, and in doing so represents the first attempt to gauge widespread public opinion on EU emergency politics.^3^ It proceeds with reference to a recent, salient and common crisis: Covid-19. The pandemic has already been located by emergency politics scholars as a continuation of the crisis-ridden 2010s, firmly restating this now familiar mode of policymaking at the start of the new decade (White 2019; Kreuder-Sonnen and White 2022; Heupel et al. 2021; Schmidt 2022). Yet, drawing on new survey data on Covid emergency politics from 15 diverse EU states, we find little suggestion that this is further alienating the public. Overall, we identify limited levels of concern across multiple measures of emergency politics and suggest that transnational cleavage theory is a more consistent identifier of citizens’ priors, pointing to socio-economic and attitudinal determinants that shape interpretations. Even accounting for different attitudes towards democracy, ‘national-communitarian’ Eurosceptics appear more concerned with emergency politics than pro-European ‘cosmopolitan-universalists’. On this initial empirical enquiry, emergency politics are not observed as driving Euroscepticism, rather the opposite: attitudes towards European integration shape evaluations of crisis management. This is not to say that EU emergency politics is not prevalent or in some way democratically corrosive, but rather that the EU’s procedural management of the salient Covid-19 case cannot readily be linked to this corrosion via immediate spikes in regularised measures of Euroscepticism. This matters, both for the ongoing scholastic debate over emergency politics, but also for those seeking to understand the sources of Euroscepticism. While it surely cannot be argued that crisis-fighting in permanence is good for the EU’s public image, equally this might not be as damaging as emergency politics scholars imply.

The article has five sections. We start by surveying the recent transnational emergency politics literature before juxtaposing it with transnational cleavage-based accounts. We then operationalise emergency politics in four dimensions with references to specific facets of leading contemporary scholarship. Third, we describe our survey data and methodology to probe the determinants of individuals’ perceptions of the four dimensions. Fourth, we show results in two stages: a brief aggregate overview of national trends, followed by individual-level data. We conclude with a discussion of results, possible limitations and by inviting further research with useful alternative designs and data.

## Transnationalism and the EU: two theoretical approaches

### Emergency politics

The spectre of permanent crisis is said to haunt the EU. But this is possibly not entirely accidental. Contemporary scholars of European emergency politics have suggested that national and transnational executives may cultivate or exploit crises, utilising their threat or actual occurrence to accrue powers, and build institutions to permanently scan horizons for further extraordinary challenges (Rhinard 2019). While appeals to exceptional challenges are ephemeral, their effect on executive power is potentially lasting, bypassing and transforming established democratic norms and procedures. This corrodes the EU’s deliberative functions as emergency governance shifts from established venues often in member states—parties, parliaments, cabinets—to the transnational European level, where power is hard to locate (White 2019). This is a more strident critique than highlighting the EU’s ‘democratic deficit’ (Follesdal and Hix 2006), because it argues that formal democratic institutions—flawed though they may be—are no bulwark against the routinisation of exceptional appeals for emergency powers. In the EU, exceptions appear to be occurring with such regularity as to resemble the norm. Scholars depart on the extent to which emergency politics might be legitimised (cf. White 2019; Kreuder-Sonnen 2019), but they share essential concerns for how extraordinary interventions are rhetorically defended and normalised without recourse to established democratic institutions (Schmidt 2022).

Kreuder-Sonnen and White (2022, 956) argue that even though novel, the Covid-19 pandemic exhibited familiar traits recurrent over the past decade. Domestic and transnational emergency politics reinforced each other in unprecedented ways: “[T]he coronavirus crisis has escalated this type of exceptionalism across the Union. Many governments have imposed the greatest restrictions on civil rights and liberties in their countries since World War II. Importantly, while domestic emergency politics may seem exclusively domestic, it too has transnational features” (Kreuder-Sonnen and White 2022, 959; cf. Schmidt 2022; Heupel et al. 2021).

Interdependence in the EU creates political transnationalism: expertise is agreed upon, each government’s action has effects on others, comparisons are made between member states’ performance, and so on. This transnationalism should have clear implications for the public’s frame of reference, but the widespread identification of novel and problematic transnational emergency politics running through Europe’s recent crises are not backed up with scrutiny of public opinion. Emergency politics scholarship is, by definition, concerned with action originating on the supply-side of policymaking.^4^ As such, executive politics have been the focus of recent emergency politics studies. The literature itself is clear that executives at the national and EU levels must contend with public opinion in democratic polities, but it is surprisingly uninterested in whether voters—be they confronted with a contagious disease or a financial panic—actually prefer swift action to routine democratic contestation. At worst, these accounts implicitly depict actors on the demand-side of policymaking processes—the public and even national parliamentarians—as passive or disempowered recipients of a form of ‘authoritarian managerialism’ (Joerges and Glinski 2014) and ‘executive dominance’ that bypasses formal mediating representative institutions such as legislatures (Griglio 2020).

This line of reasoning risks becoming circular if perceptions and drivers of demand are not studied independently. White (2019, ch. 6) offers a partial corrective by theorising an explicit link between emergency politics and the rise of nationalist populism, which he labels the ‘emergency-to-come’. He sketches out a plausible account of creeping disaffection that has been ripe for exploitation during the EU’s long decade of crisis. Here, emergency politics amounts to a ‘disavowal of agency’ by political representatives, who follow the alleged imperatives of a crisis situation in which there is no time for deliberation nor space for alternatives. Populist leaders should be taken seriously insofar as they resist the transnational orchestration of problem-solving, delivering—rhetorically at least—the ‘promise of agency’ to the masses (White 2019).

An alternative account underlines that citizens whose livelihoods are threatened by contagious disease or market panic expect policies that are commensurate to these threats. In the terminology of Scharpf (2009) and Schmidt (2013), the emergency politics literature implicitly postulates that citizens should care about input and throughput legitimacy: the majoritarian, deliberative and procedural validation of authoritative decision-making. However, what if in crisis, voters are disproportionately concerned about the output legitimacy provided by effective policymaking? Vivien Schmidt’s (2022, 990) reading of recent EU emergency politics acknowledged this possibility: “In cases where [emergency] practices appeared to ensure output performance and were rhetorically legitimated by agents whose discourse deployed persuasive power through ideas, emergency actions tended to be normalised, even where input responsiveness or throughput quality initially appeared to be in short supply.” Voters’ disempowerment is also not total or ubiquitous. Kreuder-Sonnen (2019) identifies a ‘ratchet effect’ where emergency powers may be normalised after the fact, but also a ‘rollback effect’ where public backlashes force international organisations to cede temporary powers.

Such conclusions qualify the claim that emergency politics can only have detrimental effects on the public’s agency and the accountability of policymakers. Like any political intervention, if emergency politics can be persuasively justified, publics may accept it, not because they are resigned to the imposition of such measures but because they can see their rationale. This creates latitude for leaders to act in a manner whereby swift action may demonstrate agility, or a form of democratic responsiveness that, in turn, may raise awareness that democracy needs engagement (Honig 2009). In this formulation, emergency politics complements accounts of ‘stealth democracy’ (Hibbing and Theiss-Morse 2002; Webb 2013), according to which many voters are not interested in the process of policymaking and do not seek greater participation in decision-making. Notably, Webb (2013) finds that in the UK at least, stealth democrats exhibit attitudes closely associated with populism and yet reject the promise of greater political participation.

This hints at an inversion of White’s (2019) causal story: those already holding populist attitudes on Europe may accept more, not less emergency politics. Rather than seeking agency, populists may desire more decisive and less deliberative, ‘talking shop’ governance. White (2019, 140–46) is careful to qualify that emergency politics is certainly not the only road to populism, nor does he suggest populists are equipped to deliver on their promise. Yet, stealth democracy implies that populist Eurosceptics may harbour an authoritarian orientation. This can be examined further by disaggregating the literature on the new transnational cleavage.

### Socio-economic cleavages

Cleavage-based theories of political conflict in advanced industrial economies are similarly preoccupied with transnational transformations, but offer a different causal story. Here, mass public sentiments are not chiefly a function but a driver of elite governance. Two influential accounts, both drawing on Lipset and Rokkan (1967), exemplify this argument in the European context. In Kriesi et al. (2008), conflict is structurally rooted, juxtaposing the ‘losers of globalisation’ or the ‘left behind’ with the ‘winners of globalisation’ or the now familiar pejorative, ‘cosmopolitan elites’. These authors predicted that mainstream parties would adapt and absorb the discontent of losers, shifting party systems rightward. In contrast, Hooghe and Marks (2018) describe an alternative trajectory: the politicisation of European integration pits ‘cosmopolitan-universalist’ parties and their voters against ‘nationalist-communitarians’, as the distributive effects of liberal internationalisation are felt. Since programmatically inflexible parties find it difficult to respond adequately to nationalist-communitarian preferences, challenger parties emerge to satisfy unmet demand.

No matter their emphasis, such readings highlight structural transformations undergirding the ways opposing socio-economic groups interpret politics. Their findings suggest that voters on either side of the divide can be identified by characteristics including age, education, and urban–rural residence. With the increasing salience of this cleavage vis-à-vis ethno-religious and capital-labour predecessors, identity politics have become more important, constraining decision-making both at EU and domestic levels (Deutschmann et al. 2018; Kuhn 2019). Broadly, new cleavage theory is particularly sensitive to the ‘demand’ side of politics, the mass public identities and orientations that are said to structure party systems, policymaking and attitudes towards European integration from below, during normal and crisis times.^5^ These identities constrain EU policymaking.

In policy terms, the new divide chiefly concerns conflicts about immigration, competing supranational sources of authority, and international economic competition. Hooghe et al. (2002) label the protagonists, respectively, ‘Green, Alternative, Libertarian’ and ‘Traditional, Authoritarian, Nationalist’ (GAL-TAN). Importantly for this discussion, internal tensions are implied within these descriptors. GAL constituents are considered pro-transnationalism but concerned with how democracy might be “weaken[ed]” by European integration (Hooghe et al. 2002, 977). Nationalist and authoritarian traits might also lead to, respectively, conflicting perceptions of EU emergency politics: troubled by transnational distance but more tolerant of decisiveness and disregard for ‘talking shop’ deliberation.

### Hypotheses

The preceding discussion leads us to the following three broad hypotheses about the EU’s Covid emergency politics. H1 reflects White’s position that populism expresses resistance to the disempowering effects of transnational emergency politics. However distorted this expression is, it reveals a more general, latent sensitivity among voters to crisis management in permanence. White’s account of populism implies that discontent with emergency politics cuts across demographics. Hence, we would expect to see quite widespread disaffection with emergency politics in our aggregate summary findings, plus generally high levels of predicted identification (though potentially different in degree), when analysing our regression results. These should not be skewed by underlying socio-demographic or political-attitudinal variables associated with new cleavage theory.

#### H1

* Emergency Politics:* Respondents are critical of features that characterise EU emergency politics, independent of differences in political reasoning underlying their criticism.

The following two hypotheses disentangle two possible readings of the cosmopolitan-communitarian (GAL-TAN) cleavage. First, H2 expects nationalist or cosmopolitan impulses to be the dominant predictor, with the views expressed anchored in prior feelings about the EU. H3 expects views on the EU to be subsumed by prior preferences for democratic deliberation or authoritative decision-making, the libertarian-authoritarian attitudes towards democracy that further divide GAL and TAN profiles (Hooghe et al. 2002). We expect to see lower levels of overall identification with emergency politics in the aggregate and much more conditional or bi-modal discontent with particular aspects of exceptional politics if either of these hypotheses are supported.

#### H2

* - Cleavage - EU Demarcation:* Euroscepticism will be associated with more critical appraisals of emergency politics, regardless of attitudes towards democracy

#### H3

* - Cleavage - Democratic Governance:* Libertarianism will be associated with more critical appraisals of emergency politics, regardless of orientations towards European integration.

## Operationalising emergency politics

### Four dimensions of emergency politics

Having laid out the terms of debate, we operationalise emergency politics in four dimensions capturing key problematic practices of EU crisis politics according to the literature (Heupel et al. 2021, 1961; Kreuder-Sonnen and White 2022, 955). We formulated each as straightforwardly as possible, such as to be accessible to citizens not disproportionately interested in politics. These four features cover central tenets highlighted by the literature, but in the absence of empirical precedents, this is necessarily an opening account, not a comprehensive survey of all possible manifestations and sentiments.

First, *script* concerns the narrowing of policy agendas left for legitimate debate. A core claim of EU emergency politics is that it appeals to necessity and applies a one-script-for-each-crisis template to set agendas. White (2015, 312) suggests this comes easily to the EU, because “where actors are numerous and fragmented, their cooperation and compliance in question, there is a natural incentive to push policies as necessary and urgent.” But in a union of diverse member states, the coordination of public health measures and supervised implementation may therefore concern voters who feel that what suits EU institutions, a majority of member states or the powerful members, is not right for their country. Others may think that coordinated action may be well-suited to containing a contagious disease, perceiving national autonomy as unnecessary and possibly harmful.

**Script:** A common EU response to a pandemic leaves too little room for each country to devise its own strategy.

*Speed* of execution over democratic deliberation is another defining feature. Here, White (2019, 64) refers to Naomi Klein’s concept of the ‘shock doctrine’, stressing parallels between the Euro-area crisis and the “orchestrated raids” that Klein (2008, 11) sees as a feature of 'disaster capitalism'.. In a situation of an unprecedented, highly contagious disease, the case for urgency was easily made. But the proposed measures were unusually draconian and debates over supposed public health-economy trade-offs remained live. As the pandemic progressed, vaccine hesitancy, political protest and visible resistance to shutting down business and social interactions variously emerged. Hence, we juxtapose a country’s capacity to deliberate with the EU’s imperative to act.

**Speed:** When working with the EU during the pandemic, our government and national parliament could take enough time to consider different proposals.

The third dimension is *size*, where the emergency politics literature locates power asymmetries between small and large members, with EU institutions finding it more expedient to concert with large, influential member states, imposing solutions on smaller or more isolated states. White (2019, 37) claims that this is a blind spot of EU integration theories: “Intergovernmentalist accounts will tend to overlook the increasingly prominent role of the ECB and its capacity to expand its own powers, the development of the Commission as a discretionary agent, as well as the increasingly unequal status of member states.” Evidence for complaints by elites in smaller member states is not hard to find, notably against the Franco-German alliance, or by Central Eastern European members against the dominance of large Western countries (Santana et al. 2020). Equally, small states have shown themselves very capable of frustrating larger states’ preferences during critical moments of EU development (Finke 2009; Golub 2012), so voters in both state types might be critical. To guard against targeting undue influence in only either small or large states as we include both in our 15-country fieldwork, this dimension refers to ‘fair balance’.

**Size:** When the EU has responded to the pandemic, there has been a fair balance between the influence of smaller and larger member states.

Fourth, emergency politics is said to deliberately obscure *clarity* concerning where ultimate decision-making takes place and whether an elected government bears responsibility for it. “Informal and hastily improvised coordination across multiple sites of executive power, both at the supranational level and at the level of national and sub-national institutions, is one of the hallmarks of emergency rule in the transnational setting” (White 2019, 133). Abandoning the highly formalised procedures of the Community Method in the name of decisive intervention presumably leads to an absence of policy choices and of accountability for these choices. Ultimately, responsibility for health measures rested at the national level but was taken after intensive discussion at the EU level. Citizens could have justifiably different, potentially clouded, views on who was making decisions and at what level. Though a rigorous multi-level assessment is beyond the scope of this article, the formulation of this dimension also allows us to compare potential perceptions of ‘supranational’ and ‘domestic’ emergency politics in action. Unlike the others, it eschews any potential tension between EU decision-making and member state democracy, allowing for answers purely based on the perceived clarity of national decision-making.

**Clarity:** How clear is it to you who decides public health measures in [COUNTRY], such as the obligation to wear face masks and to practice social distancing?

## Data and results

### Survey design

Our survey was fielded in 15 EU countries between 24 May and 19 October 2021.^6^ We first minimally primed respondents with a basic statement about the EU’s ‘common response’ to Covid-19,^7^ before posing the four questions which collectively comprise our dependent variable of emergency politics identification (see Table A1, Appendix). As described above, we used a mix of critical and affirmative statements plus a construct-specific statement to try to mitigate against acquiescence response bias or priming effects (Pasek and Krosnick 2010, 38–39), but scaling is standardised for the following discussion.^8^

### Aggregate trends: dimensions and countries

**Fig. 1:**
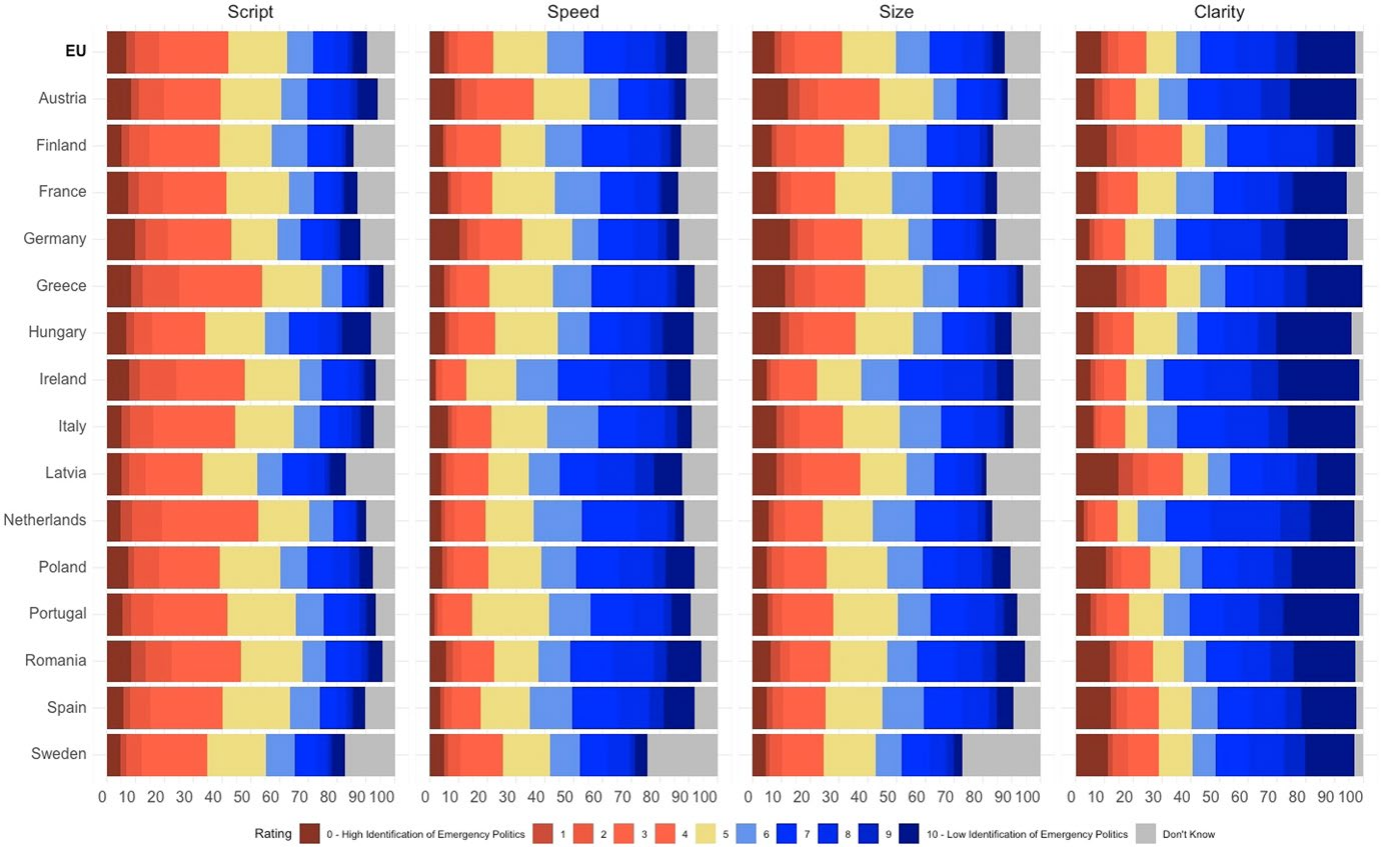
Share of responses by dimension and country. *Note*: Scores for ‘Script’ are inverted for comparability

First, we explore descriptive results at the dimension and country level. The top row of Fig. 1 depicts the share of distribution of all responses (N = 11,826). A few trends are immediately apparent. An overly uniform *Script* is the area of greatest concern with a comparatively low mean response of 4.95. Respondents disproportionately indicated a negative (red) identification of a feature of emergency politics, an overly uniform EU script. 42% agree with the statement while 28% disagree, and a significant proportion indicated a neutral or ‘Don’t Know’ response (30%). At the other end, respondents appear most likely to perceive the source of decisions as *clear* with blue bars indicating no support for this allegation of disempowerment by emergency politics (mean = 6.93). Clarity, the dimension most closely calibrated towards national politics is the only one where an outright majority falls on one side of the argument, with 62% of respondents overall indicating that Covid-19 decision-making was clear (25% negative). Undue *Speed* (6.07) and asymmetric influence of *Size* (5.53) fall in-between: there is not much evidence for widespread perceived haste (48% agree, 22% negative); as regards the fair balance between differently-sized member states the vote is split (37% agree, 31% disagree). Levels of neutrality or uncertainty also vary. While only 13% of respondents answered either a neutral 5 or ‘Don’t Know’ for the Clarity dimension, this measure of uncertainty increases to 30% for Script and Speed, 31% for Size.

Figure 1 also shows national trends. There are no strong outliers and a majority or plurality of identification with emergency politics is only present in a handful of the state-dimension combinations. But even where the perception of an overly uniform Script was stronger than average, notably in Greece and the Netherlands, this did not extend to the other dimensions in favour of perceived emergency politics. On Speed, in Austria and Germany, more respondents felt that decision-making was too hasty and the same two countries also score above average on Clarity of decision-making at home. As regards Size, it is noticeable that respondents in some smaller member states like Ireland and Romania seem less concerned about power asymmetry than the EU-average, while Germany and Greece score similarly highly. It appears that in addition to not being an overriding concern at the EU level, there is no consistent country-based trend to speak of here, rather pockets of national concern about certain dimensions.

### Individual determinants

The aggregate data cast some doubt on strong claims of emergency politics but cannot assess cleavage theory. It leads us to expect sizeable, if not necessarily equal, shares for agreement and disagreement with statements or clear/unclear answers, respectively. The red and blue bars in Fig. 1 appear compatible with this conjecture. But we need to know whether respondents and characteristics are associated as predicted by cleavage theory. Accordingly, socio-economic and demographic characteristics should associate with profiles of political identity, either pro-integration (GAL: Green, Alternative, Libertarian) or pro-demarcation (TAN: Traditional, Authoritarian, Nationalist). The latter are also overwhelmingly older, less formally educated, rural and/or expressing a strong national identity (Hooghe and Marks 2018). If significant, these predictors might offer indications as to whether perceptions of emergency politics are filtered through respondents’ broader views on European integration and democratic politics. Table 1 summarises controls, scales and their relations to our hypotheses.

**Table 1:** Individual determinants of emergency politics perceptions in cleavage theory

Variable	Categories (OLR reference in bold)	Expected association with emergency politics identification
Identity	**European Only**, European and [Country Nationality], [Country Nationality] and European, [Country Only]	+ National identity
Age	**18**–88	+ Age
Education	**Low** (≤ 15), Middle (16–19), High (≥ 20)	− Education
Residence	**Rural area or village**, Small to mid-sized town, Large town or city	+ Ruralness
Ideology	**Left (0)–**Right (10)	+ Right
European integration	**EU integration has gone too far (0)**, EU integration should be pushed further (10)	+ Hostility to EU integration (H2)
Government preferences	**Democracy always preferable**, Authoritarian government sometimes preferable, Indifference	+ Attachment to democracy (H3)
Leadership preferences	Good to have a strong leader who does not bother with parliaments and elections (**0—Completely Disagree**, 10 Completely Agree)	+ Attachment to democracy (H3)

**Fig. 2:**
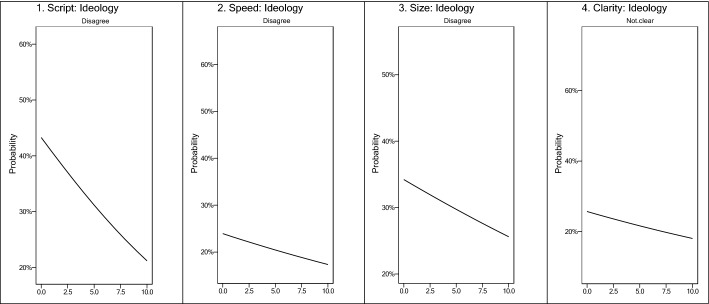
Predictions from ordered logit models 1, 4, 7, 10 (Table 2)

We estimate ordered logit regression (OLR) models using the determinants described in Table 2 to see whether they can predict attitudes towards emergency politics in line with two possible readings of cleavage theory, as formulated in H2 and H3. OLR models are particularly suitable for ordered categorical data with more than two response categories, like our 11-point scales (Long 1997). We present log odds regression coefficients in Table 2, which show increases or decreases in likelihood of identifying the four different elements against a baseline reference for each variable. Calculating exponentiated predictive probabilities from the models allows us to visually convey the likelihood of a respondent perceiving a measure of emergency politics given a statistically significant relationship with a predictor variable (see e.g. Fig. 2 below).

**Table 2:** Regression models—individual characteristics and emergency politics perceptions

	Dependent variable											
	Script			Speed			Size			Clarity		
	(1)	(2)	(3)	(4)	(5)	(6)	(7)	(8)	(9)	(10)	(11)	(12)
ID: EU > Country	− 0.06	− 0.03	− 0.06	0.08	0.09	0.08	0.06	0.05	0.06	0.17	0.19	0.17
	(0.11)	(0.11)	(0.11)	(0.12)	(0.12)	(0.12)	(0.11)	(0.12)	(0.11)	(0.12)	(0.12)	(0.12)
ID: Country > EU	− 0.05	− 0.04	− 0.05	− 0.09	− 0.09	− 0.09	− 0.07	− 0.10	− 0.07	0.19*	0.20*	0.19*
	(0.11)	(0.11)	(0.11)	(0.11)	(0.12)	(0.11)	(0.11)	(0.11)	(0.11)	(0.11)	(0.12)	(0.11)
ID: Country Only	0.12	0.15	0.11	− 0.40***	− 0.37***	− 0.41***	− 0.34***	− 0.36***	− 0.34***	− 0.03	0.03	− 0.03
	(0.12)	(0.12)	(0.12)	(0.12)	(0.13)	(0.12)	(0.12)	(0.12)	(0.12)	(0.12)	(0.13)	(0.12)
ID: None	− 0.04	0.04	− 0.04	− 0.53***	− 0.47***	− 0.53***	− 0.56***	− 0.59***	− 0.56***	− 0.38**	− 0.35**	− 0.37**
	(0.16)	(0.17)	(0.16)	(0.17)	(0.18)	(0.17)	(0.17)	(0.17)	(0.17)	(0.17)	(0.18)	(0.17)
Age	− 0.003**	− 0.003**	− 0.003**	0.003**	0.001	0.003**	− 0.001	− 0.001	− 0.001	0.003**	0.001	0.003**
	(0.001)	(0.001)	(0.001)	(0.001)	(0.001)	(0.001)	(0.001)	(0.001)	(0.001)	(0.001)	(0.001)	(0.001)
Edu: Middle	− 0.15**	− 0.17**	− 0.15**	0.08	0.05	0.08	− 0.12*	− 0.15**	− 0.12*	0.13*	0.08	0.13*
	(0.07)	(0.07)	(0.07)	(0.07)	(0.07)	(0.07)	(0.07)	(0.07)	(0.07)	(0.07)	(0.08)	(0.07)
Edu: High	− 0.22***	− 0.25***	− 0.22***	0.16**	0.11	0.16**	− 0.05	− 0.07	− 0.05	0.11	0.02	0.11
	(0.07)	(0.07)	(0.07)	(0.07)	(0.07)	(0.07)	(0.07)	(0.07)	(0.07)	(0.07)	(0.08)	(0.07)
Town	− 0.03	− 0.03	− 0.03	0.04	0.04	0.04	− 0.02	− 0.02	− 0.02	0.04	0.02	0.04
	(0.06)	(0.06)	(0.06)	(0.06)	(0.06)	(0.06)	(0.06)	(0.06)	(0.06)	(0.06)	(0.07)	(0.06)
City	− 0.03	− 0.03	− 0.03	0.03	0.04	0.03	− 0.04	− 0.06	− 0.04	0.04	0.03	0.04
	(0.06)	(0.06)	(0.06)	(0.06)	(0.06)	(0.06)	(0.06)	(0.06)	(0.06)	(0.06)	(0.06)	(0.06)
Ideology	0.10***	0.10***	0.10***	0.04***	0.04***	0.04***	0.04***	0.04***	0.04***	0.05***	0.05***	0.05***
	(0.01)	(0.01)	(0.01)	(0.01)	(0.01)	(0.01)	(0.01)	(0.01)	(0.01)	(0.01)	(0.01)	(0.01)
EU Integration	0.002	− 0.003	0.003	0.11***	0.11***	0.11***	0.14***	0.13***	0.14***	0.09***	0.08***	0.09***
	(0.01)	(0.01)	(0.01)	(0.01)	(0.01)	(0.01)	(0.01)	(0.01)	(0.01)	(0.01)	(0.01)	(0.01)
Govt: Auth		0.10*			− 0.08			− 0.11*			− 0.35***	
		(0.06)			(0.06)			(0.06)			(0.06)	
Govt: Indif		− 0.21*			− 0.37***			− 0.24**			− 0.53***	
		(0.11)			(0.11)			(0.11)			(0.11)	
Strong Leader			0.01***			0.003			0.0003			− 0.001
			(0.002)			(0.002)			(0.002)			(0.002)
Observations	7617	7298	7617	7483	7163	7483	7401	7087	7401	7870	7539	7870

^*^p < 0.1; **p < 0.05; ***p < 0.01

1. Script: A common EU response to a pandemic leaves too little room for each country to devise its own strategy

2. Speed: When working with the EU during the pandemic, our government and national parliament could take enough time to consider different proposals

3. Size: When the EU has responded to the pandemic, there has been a fair balance between the influence of smaller and larger member states

4. Clarity: How clear is it to you who decides public health measures in [COUNTRY], such as the obligation to wear face masks and to practice social distancing?

For each dimension of emergency politics (Script, Speed, Size and Clarity), we present three models. The baseline model includes predictors for identity, age, education, residence, ideology and preferences on European integration, to test H2 (attitudes towards the EU anchor views). For H3, the second model adds the predictor on governmental preferences (democracy vs. autocracy) and the third adds leadership preferences, whether respondents agree that strong leadership that bypasses formal political institutions and checks is sometimes preferable.

Table 2 suggests several significant results. National identity has a significant and relatively strong effect on two dimensions of emergency politics: Speed and Size. That is, compared to those expressing a European identity, respondents who identify only with their country are less likely to agree that their government had enough time to consider different proposals during the coordinated EU response to Covid-19. Extrapolating predicted probabilities from the raw log odd coefficients, respondents with an exclusively national identity are expected slightly above 50% of the time to agree with the claim that national governments had enough time to consider alternatives, versus higher than 60% for those identifying as Europeans. Those with a national identity are also more likely to think that country size influence was unbalanced (35% vs. 27%). Respondents without a preference, indicating either disaffection or an unspecified alternate identity, were also significantly more likely to be critical of Covid-19 emergency politics. National or cosmopolitan identity thus seems to have a differentiated impact on views of Speed and Size, with the direction of the significant effects reinforcing the transnational reading of the GAL-TAN divide (H2), and contradicting H3’s expectations that libertarian attitudes would be associated with a greater concern for democratic governance.

However, ageing has a significant and substantive effect associated with less hostile responses on three dimensions: Script, Speed and Clarity. Again, this comparatively affirmative stance among older respondents is unexpected by the transnational precepts of cleavage theory, which would assume heightened Euroscepticism. In this specific crisis, there is a possibility that increased risks from exposure to the virus increased tolerance for executive actions and attenuates democratic concerns. More education is also significant. Middle education is associated with more tolerance of the Script and Size dimensions of emergency politics. Higher education levels are associated with a greater likelihood of agreement that alternatives to the EU script were possible and enough time was devoted to debate. Again, the transnationalist dimension of the GAL-TAN divide appears to be a stronger predictor than democratic attitudes, lending further support to H2.

This is confounded somewhat when ideology is taken as a crude alternate measure of GAL-TAN placement. Table 2 shows that it is also confirmed to play a significant, if small, role in emergency politics perceptions. Figure 2 shows that respondents who placed themselves further to the right are more likely to agree that the coordinated European response left too little room for nationally bespoke strategies (Script), while they were also more likely to agree that there was enough time to take decisions (Speed) and that there was a fair balance between the influence of large and small countries in the EU (Size). Right-wing citizens were more likely to see clear responsibility for decisions on health measures. This pattern of perceptions by right-wing respondents corresponds more closely to the predictions of cleavage theory (H3) while the finding for Clarity contradicts cleavage theory. A mirror image emerges for left-wing voters: they were less concerned about the Script aspect of emergency politics and more so by the Speed and Size of the EU’s crisis management.

Finally, we look for evidence indicating whether identifying more explicitly with Euroscepticism and authoritarianism makes respondents more or less sensitive to (certain features of) emergency politics. Respondents who think EU integration has gone too far are more likely to identify Speed, Size and Clarity (but not Script) as problematic dimensions. This finding is in line with H2 that the view of European integration will colour respondents’ perceptions generally and specifically that Eurosceptic voters are more concerned about the role of the national government in decision-making. Adding democratic versus authoritarian preferences, we find that these preferences are significant, if small, predictors and suggest that those who prefer more authoritarian types of government are more concerned about emergency politics (Table 1, Appendix). Models with preferences for strong leadership have significant coefficients for the Script dimension: respondents who agree that “it is good to have a strong leader” tend also to agree that the EU left too little room for national strategies to fight the pandemic (Table 2 and Appendix). Though the effect size is small, this lends further support to H2 and hints that democratic orientations might be subsumed by an overriding preference for national politics among TAN profile voters.

In sum, we find that significant predictors for concerns about certain aspects of emergency politics are identity, age, education, ideology, views on EU integration and attitudes towards democracy. These are mostly the determinants that should matter according to cleavage theory. However, GAL voters seem to be less critical of emergency politics than H3 expected across the board. The notion that emergency politics captures a widespread, if latent source of voter discontent and alienation is also not supported by our findings that the typical pro-demarcation TAN voter finds only some of its alleged features problematic.

## Conclusions

The claim that the EU’s extensive and prolonged crisis management has corrosive effects on democratic processes is both serious and increasingly prevalent. Scholars of EU transnational emergency politics derive from these pathologies negative associations with public opinion, suggesting that this mode of politics might give rise to nationalist populist and anti-system politics (Kreuder-Sonnen and White 2022, 691). This is a plausible but empirically under-researched argument, which this paper sought to address in two ways. First, it juxtaposed the emergency politics rendering of EU transnationalism with established and empirically-grounded work on Euroscepticism from the new cleavage theory, highlighting their inverse claims of cause and effect. Then, it operationalised emergency politics and gauged public opinion in 15 states with reference to a recent, singularly symmetrical and salient crisis for the entire EU: Covid-19.

Though far from a comprehensive test, results offer initial indications that citizens interpret emergency politics, or crisis ‘throughput’, via their prior views on the EU. Aggregate and national breakdowns of our survey results generally revealed low levels of concern, but not uniformly so. Our operationalisation of emergency politics into four dimensions (Script, Speed, Size, Clarity) shows that neither a majority of the public nor an identifiable group of pro- and anti-integrationist voters consistently perceived EU emergency politics during Covid-19 to be troubling. However, the dimension most aligned with domestic politics vis-à-vis EU influence, Clarity, does consistently show the lowest identification of problematic emergency politics.

Integration-demarcation preferences are most consistently associated with significant differences across the four dimensions. The typical citizen with a national-communitarian orientation is more critical of these dimensions than their cosmopolitan-universalist counterpart. This suggests that prior attitudes structure interpretations of emergency politics in action. Furthermore, within the GAL-TAN subgroups, we also consistently find that outlooks towards the EU appear prior to any demarcation over democracy. If TAN citizens are stealth democrats, any enthusiasm for executive leadership at the expense of democratic norms does not extend to the EU.

These findings cast doubt on the claim that crisis management might be fostering Euroscepticism. They hint at a reverse causality: attitudes towards openness and European integration override or reinforce concerns regarding crucial features of EU crisis management. This article alone cannot provide a definitive account of how crisis politics affects public opinion about transnational politics. Further research might experimentally test how post-hoc rationalisations affect public perceptions of executive rule, and whether crisis management is having a longer-term corrosive impact on perceptions of EU governance alongside or instead of the democratic deficit. Our confounding result that older respondents showed greater tolerance of Covid emergency politics clearly prompts the interrogation of a wider set of crisis cases. It is also necessary to ask whether the notion of a cleavage leaves out those citizens who neither identify consistently as GAL nor TAN, and do not hold strong views on integration-demarcation. Which concerns shape their views of EU crisis management and how do they react to its politicisation? Are this important group ‘stealth democrats’ or might they be alienated by EU crisis management? The emergency politics literature has placed such concerns firmly onto the research agenda of advanced democracies and experimental polities existing in a now apparent state of permanent crisis.

## Supplementary Information

Below is the link to the electronic supplementary material.

Supplementary file1 (DOCX 798 KB)

## Footnotes

1 For a recent review including multiple leading scholars working in this tradition, see Heupel et al. (2021).

2 We thank Joan Miró for this expression.

3 But see Bertsou and Caramani (2022) for a related attempt to evaluate public opinion of experts.

4 For instance, even though White explores the attractiveness of populism for voters, he stresses the ‘supply-side’ character of the phenomenon: ‘[…] the politics of “populism” is propelled at least as much by the weaknesses of representative democracy under conditions of emergency rule as by any popular attraction to the specific features one tends to associate with it (non-pluralism, anti-elitism, etc.). In the terms of the debate, it is a problem of supply rather than demand.’ (White 2019, 143).

5 This is to present a somewhat stylised juxtaposition. The literature on a new transnational cleavage in domestic politics has been complemented by important contributions on ‘supply side’ populism (see e.g. Hobolt and De Vries 2020). But even with this complement, cleavage theory starts with the response of changing public demand to underlying socio-economic transformations to which supply responds more or less pro-actively, while emergency politics accounts start with elite policymaking in the mode of crisis management that offers the voting public more or less essential choice.

6 More detailed information on survey design can be found in the Appendix.

7 The statement preceding the four questions reads: “The EU had a common response to the COVID-19 pandemic, for example centrally ordering medical equipment, including vaccines, and creating the Recovery Fund. To what extent do you agree or disagree with the following statements?”.

8 Negative (script), positive (speed, size) and construct-specific (clarity). Though we do notice generally more acquiescence for speed and size than script, we find the most positive (non-emergency politics identifying) responses in clarity. This indicates that any present acquiescence effect was likely minimal.
