# Supplementary material for: Who is afraid of emergency politics? Public opinion on European crisis management during Covid-19
Source: Comp Eur Polit. 2023 Feb 6:1–21. Online ahead of print. doi: 10.1057/s41295-023-00329-5 (PMC9899656; doi:10.1057/s41295-023-00329-5)
Supplement: Supplementary file 1 — Supplementary file1 (DOCX 798 KB) [file 41295_2023_329_MOESM1_ESM.docx]

**Appendix – Survey Information**

The survey data used in this paper were drawn from part of a more extensive survey on recent EU crisis politics, covering 16 European countries (UK respondents were not surveyed on aspects of EU emergency politics). Fieldwork was conducted mostly in June 2021, with trials and quota balancing supplementary samples conducted in May and October 2021 respectively (see Table A1). These countries were selected primarily for their exposure to or direct involvement in one or more major EU crisis events over the past decade, but also to ensure regional and size-based variation. Owing to budgetary and sampling limitations, unfortunately it was not possible to survey all 28 states. The lengthy and demanding nature of the survey meant that each respondent only answered two of five in-depth modules, including the Covid Crisis module containing the four dimensions of emergency politics.

- **Vendor:** Gallup Europe
- **Countries (Available Languages):**
  - Austria (German)
  - Finland (Finnish, Swedish)
  - France (French)
  - Germany (German)
  - Greece (Greek)
  - Hungary (Hungarian)
  - Ireland (English)
  - Italy (Italian)
  - Latvia (Latvian, Russian)
  - Netherlands (Dutch)
  - Poland (Polish)
  - Portugal (Portuguese)
  - Romania (Romanian)
  - Spain (Spanish)
  - Sweden (Swedish)
  - UK (English)
- **Fieldwork:** 16-29/30 June 2021 in all countries unless stated (see Table A1)
- **Data Collection:** CAWI (Online only)
- **Respondents Per Country:** 2000 (32,000 Total)
- **Weighting Variables:** Gender/Age/Education/Rural-Urban Location
- **Attention Checks:** No
- **Median Response Time (rounded minutes):** 21
- **Question Modules (All Respondents):**
  - Socio-Demographics
  - Political Attitudes
  - EU Crisis Attitudes (Comparative)
- **In-Depth Crisis Modules (40% of respondents per module, random ordering):**
  - ***Covid Crisis (Emergency Politics Specific Questions)***
  - Membership Crisis
  - Social Crisis
  - Migration Crisis
  - Eurozone Crisis
- **Quota-Modulation:** Each respondent receives 2 of 5 in-depth crisis modules, ordered randomly

**Table 1A. Summary Statistics – Countries and Dependent Variables**

|  | Total  Resps. | Supplementary  Fieldwork (No. Respondents) | Median Response (Mins) | Script | | | | Speed | | | | Size | | | | Clarity | | | |
| --- | --- | --- | --- | --- | --- | --- | --- | --- | --- | --- | --- | --- | --- | --- | --- | --- | --- | --- | --- |
|  |  |  |  | Mean | Mode | St Dev | DKs | Mean | Mode | St Dev | DKs | Mean | Mode | St Dev | DKs | Mean | Mode | St Dev | DKs |
| AT | 745 |  | 23 | 5.2 | 5 | 2.7 | 6.0 | 4.8 | 5 | 2.6 | 11.0 | 4.2 | 5 | 2.6 | 11.3 | 7 | 10 | 3.0 | 2.4 |
| DE | 705 |  | 21 | 5.5 | 5 | 2.8 | 11.8 | 4.9 | 5 | 2.8 | 13.5 | 4.5 | 5 | 2.8 | 15.5 | 7 | 10 | 2.8 | 5.5 |
| ES | 847 | 5 Oct - 18 Oct (80) | 21 | 5.4 | 5 | 2.3 | 10.2 | 6.2 | 5 | 2.5 | 8.0 | 5.5 | 5 | 2.4 | 9.4 | 6 | 10 | 3.3 | 2.5 |
| FI | 741 |  | 21 | 5.4 | 5 | 2.3 | 14.3 | 5.6 | 7 | 2.4 | 12.7 | 4.9 | 5 | 2.5 | 16.5 | 5 | 8 | 3.1 | 3.0 |
| FR | 731 | 5 Oct - 16 Oct (4) | 21 | 5.6 | 5 | 2.5 | 12.9 | 5.4 | 5 | 2.5 | 13.5 | 5.0 | 5 | 2.5 | 15.2 | 6 | 10 | 2.9 | 5.7 |
| GR | 964 | 5 Oct - 15 Oct (141) | 23 | 5.9 | 5 | 2.5 | 4.0 | 5.7 | 5 | 2.4 | 8.1 | 4.6 | 5 | 2.6 | 6.0 | 6 | 10 | 3.4 | 0.5 |
| HU | 837 | 5 Oct - 18 Oct (46) | 25 | 4.8 | 5 | 2.8 | 8.1 | 5.8 | 5 | 2.6 | 8.5 | 4.8 | 5 | 2.7 | 10.0 | 7 | 10 | 3.1 | 4.3 |
| IR | 744 | 24 May - 25 May (16) | 22 | 5.7 | 5 | 2.5 | 6.7 | 6.4 | 7 | 2.2 | 9.4 | 5.8 | 7 | 2.5 | 9.4 | 7 | 10 | 2.9 | 1.6 |
| IT | 689 |  | 20 | 5.3 | 5 | 2.4 | 7.1 | 5.6 | 5 | 2.4 | 9.1 | 5.0 | 5 | 2.5 | 9.4 | 7 | 10 | 2.8 | 2.8 |
| LV | 826 |  | 28 | 5.0 | 5 | 2.5 | 16.9 | 6.1 | 5 | 2.6 | 12.2 | 4.5 | 5 | 2.5 | 18.8 | 5 | 0 | 3.4 | 2.7 |
| NL | 757 |  | 21 | 5.9 | 5 | 2.2 | 9.9 | 5.7 | 7 | 2.2 | 11.8 | 5.3 | 5 | 2.4 | 16.8 | 7 | 8 | 2.5 | 3.0 |
| PL | 927 | 5 Oct - 26 Oct (154) | 23 | 5.2 | 5 | 2.6 | 7.7 | 6.0 | 5 | 2.5 | 7.9 | 5.5 | 5 | 2.5 | 10.4 | 6 | 10 | 3.3 | 2.9 |
| PT | 892 | 5 Oct - 18 Oct (91) | 25 | 5.4 | 5 | 2.3 | 6.5 | 6.0 | 5 | 2.1 | 9.3 | 5.3 | 5 | 2.4 | 8.1 | 7 | 10 | 2.9 | 1.5 |
| RO | 700 |  | 23 | 5.6 | 5 | 2.6 | 4.3 | 6.0 | 5 | 2.7 | 5.6 | 5.6 | 5 | 2.7 | 5.3 | 6 | 10 | 3.3 | 2.7 |
| SE | 721 |  | 22 | 5.2 | 5 | 2.4 | 17.3 | 5.2 | 5 | 2.5 | 24.4 | 5.1 | 5 | 2.4 | 27.3 | 6 | 10 | 3.2 | 3.1 |
| **EU** | **11826** | **24 May – 18 Oct** | **22** | **5.4** | **5** | **2.5** | **9.6** | **5.7** | **5** | **2.5** | **11** | **5.1** | **5** | **12.6** | **6.3** | **9.1** | **10** | **3.1** | **2.9** |

**Additional Plots of Ordered Logit Regressions**

| Figure 1A Predictions from Models 1, 4, 7, 10 in Table 2 | | | |
| --- | --- | --- | --- |
|  |  |  |  |
|  |  |  |  |
|  |  |  |  |
|  |  |  |  |
|  |  |  |  |
| Figure 2A - Predictions from Models 2, 5, 8, 11 in Table 2 | | | |
|  |  |  |  |
| Figure 3.A Predictions from Models 3, 6, 9, 12 in Table 2 | | | |
|  |  |  |  |

Figure 5A – Country Fixed Effects by Emergency Politics Dimension (baseline: Germany)

Table 2A – Replication Regression (OLS Model)

|  | **Dependent Variable** | | | | | | | | | | | |
| --- | --- | --- | --- | --- | --- | --- | --- | --- | --- | --- | --- | --- |
|  | Script | | | Speed | | | Size | | | Clarity | | |
|  | (1) | (2) | (3) | (4) | (5) | (6) | (7) | (8) | (9) | (10) | (11) | (12) |
| ID: EU>Country | -0.15 | -0.13 | -0.11 | 0.07 | 0.07 | 0.12 | 0.21 | 0.20 | 0.23^*^ | 0.09 | 0.13 | 0.09 |
|  | (0.14) | (0.14) | (0.13) | (0.13) | (0.13) | (0.13) | (0.14) | (0.14) | (0.14) | (0.16) | (0.17) | (0.17) |
|  |  |  |  |  |  |  |  |  |  |  |  |  |
| ID: Country>EU | -0.10 | -0.10 | -0.08 | -0.13 | -0.14 | -0.11 | -0.02 | -0.05 | -0.01 | 0.17 | 0.20 | 0.17 |
|  | (0.13) | (0.13) | (0.13) | (0.12) | (0.13) | (0.12) | (0.13) | (0.13) | (0.13) | (0.16) | (0.16) | (0.16) |
|  |  |  |  |  |  |  |  |  |  |  |  |  |
| ID: Country | 0.09 | 0.12 | -0.02 | -0.48^***^ | -0.44^***^ | -0.54^***^ | -0.46^***^ | -0.45^***^ | -0.53^***^ | -0.20 | -0.08 | -0.22 |
|  | (0.14) | (0.15) | (0.14) | (0.14) | (0.14) | (0.14) | (0.14) | (0.15) | (0.14) | (0.17) | (0.18) | (0.17) |
|  |  |  |  |  |  |  |  |  |  |  |  |  |
| ID: No | 0.001 | 0.06 | 0.01 | -0.64^***^ | -0.63^***^ | -0.63^***^ | -0.76^***^ | -0.80^***^ | -0.72^***^ | -0.66^***^ | -0.57^**^ | -0.68^***^ |
|  | (0.20) | (0.21) | (0.20) | (0.19) | (0.20) | (0.20) | (0.20) | (0.21) | (0.20) | (0.24) | (0.25) | (0.25) |
|  |  |  |  |  |  |  |  |  |  |  |  |  |
| Age | -0.004^***^ | -0.004^**^ | -0.003^*^ | 0.005^***^ | 0.003^**^ | 0.01^***^ | -0.001 | -0.002 | -0.0004 | 0.01^***^ | 0.003 | 0.01^***^ |
|  | (0.002) | (0.002) | (0.002) | (0.002) | (0.002) | (0.002) | (0.002) | (0.002) | (0.002) | (0.002) | (0.002) | (0.002) |
|  |  |  |  |  |  |  |  |  |  |  |  |  |
| Edu: Middle | -0.21^**^ | -0.24^***^ | -0.13 | 0.10 | 0.07 | 0.14^*^ | -0.14^*^ | -0.18^**^ | -0.12 | 0.09 | 0.02 | 0.08 |
|  | (0.08) | (0.09) | (0.08) | (0.08) | (0.08) | (0.08) | (0.08) | (0.09) | (0.08) | (0.10) | (0.10) | (0.10) |
|  |  |  |  |  |  |  |  |  |  |  |  |  |
| Edu: High | -0.30^***^ | -0.33^***^ | -0.18^**^ | 0.08 | 0.04 | 0.13 | -0.12 | -0.15^*^ | -0.08 | 0.10 | -0.001 | 0.08 |
|  | (0.08) | (0.09) | (0.08) | (0.08) | (0.08) | (0.08) | (0.08) | (0.09) | (0.08) | (0.10) | (0.10) | (0.10) |
|  |  |  |  |  |  |  |  |  |  |  |  |  |
| Town | -0.10 | -0.10 | -0.09 | 0.03 | 0.03 | 0.02 | -0.03 | -0.03 | -0.03 | 0.05 | 0.01 | 0.06 |
|  | (0.07) | (0.07) | (0.07) | (0.07) | (0.07) | (0.07) | (0.07) | (0.08) | (0.07) | (0.09) | (0.09) | (0.09) |
|  |  |  |  |  |  |  |  |  |  |  |  |  |
| City | -0.14^**^ | -0.14^**^ | -0.13^*^ | 0.03 | 0.04 | 0.03 | -0.02 | -0.02 | -0.01 | 0.14 | 0.11 | 0.14^*^ |
|  | (0.07) | (0.07) | (0.07) | (0.07) | (0.07) | (0.07) | (0.07) | (0.07) | (0.07) | (0.09) | (0.09) | (0.09) |
|  |  |  |  |  |  |  |  |  |  |  |  |  |
| Ideology | 0.14^***^ | 0.13^***^ | 0.10^***^ | 0.04^***^ | 0.05^***^ | 0.03^**^ | 0.05^***^ | 0.05^***^ | 0.03^**^ | 0.06^***^ | 0.07^***^ | 0.06^***^ |
|  | (0.01) | (0.01) | (0.01) | (0.01) | (0.01) | (0.01) | (0.01) | (0.01) | (0.01) | (0.01) | (0.01) | (0.01) |
|  |  |  |  |  |  |  |  |  |  |  |  |  |
| EU Integration | -0.02 | -0.02 | -0.01 | 0.16^***^ | 0.15^***^ | 0.16^***^ | 0.18^***^ | 0.18^***^ | 0.19^***^ | 0.14^***^ | 0.13^***^ | 0.14^***^ |
|  | (0.01) | (0.01) | (0.01) | (0.01) | (0.01) | (0.01) | (0.01) | (0.01) | (0.01) | (0.01) | (0.01) | (0.01) |
|  |  |  |  |  |  |  |  |  |  |  |  |  |
| Govt: Auth |  | 0.16^**^ |  |  | -0.19^***^ |  |  | -0.22^***^ |  |  | -0.57^***^ |  |
|  |  | (0.07) |  |  | (0.07) |  |  | (0.07) |  |  | (0.09) |  |
|  |  |  |  |  |  |  |  |  |  |  |  |  |
| Govt: Indif. |  | -0.17 |  |  | -0.37^***^ |  |  | -0.27^**^ |  |  | -0.70^***^ |  |
|  |  | (0.14) |  |  | (0.13) |  |  | (0.13) |  |  | (0.16) |  |
|  |  |  |  |  |  |  |  |  |  |  |  |  |
| Strong Lead |  |  | 0.14^***^ |  |  | 0.07^***^ |  |  | 0.08^***^ |  |  | -0.01 |
|  |  |  | (0.01) |  |  | (0.01) |  |  | (0.01) |  |  | (0.01) |
|  |  |  |  |  |  |  |  |  |  |  |  |  |
| Constant | 6.43^***^ | 6.47^***^ | 5.90^***^ | 4.85^***^ | 5.03^***^ | 4.53^***^ | 4.86^***^ | 5.02^***^ | 4.56^***^ | 6.32^***^ | 6.71^***^ | 6.33^***^ |
|  | (0.22) | (0.23) | (0.22) | (0.21) | (0.22) | (0.21) | (0.22) | (0.23) | (0.22) | (0.26) | (0.27) | (0.27) |
| Country FE | Yes | Yes | Yes | Yes | Yes | Yes | Yes | Yes | Yes | Yes | Yes | Yes |
| Observations | 7,617 | 7,298 | 7,484 | 7,483 | 7,163 | 7,355 | 7,401 | 7,087 | 7,282 | 7,870 | 7,539 | 7,696 |

Note: ^*^p<0.1 ; ^**^p<0.05 ; ^***^p<0.01
